# Supplementary material for: Effects of global Ripk2 genetic deficiency in aged mice following experimental ischemic stroke
Source: Aging Brain. 2025 Mar 29;7:100135. doi: 10.1016/j.nbas.2025.100135 (PMC11993155; doi:10.1016/j.nbas.2025.100135)
Supplement: Supplementary Data 1 [file mmc1.docx]

**SUPPLEMENTARY DATA**

**Title**: Effects of Global *Ripk2* Genetic Deficiency in Aged Mice following Experimental Ischemic Stroke

**Authors**: John Aaron Howell^1,2^, Jonathan Larochelle^1,2^, Rachel E. Gunraj^1,2^, Sofia M. Stansbury^1,2^, Lei Liu^1,2^, Changjun Yang^1,2^, Eduardo Candelario-Jalil^1,2^

**Affiliations**: University of Florida ^1^Department of Neuroscience and ^2^McKnight Brain Institute

**Ripk2-/- mice spend less time in the center of the open field chamber than aged WT control mice.** At baseline, before induction of permanent middle cerebral artery occlusion, *Ripk2^-/-^* mice spent less time in the center of the open field arena than aged WT control mice. This also happened at days 1 and 3 post-stroke, with strong trends throughout the study. This could be indicative of higher anxiety-like behavior in *Ripk2*^-/-^ mice than in the WT control mice.


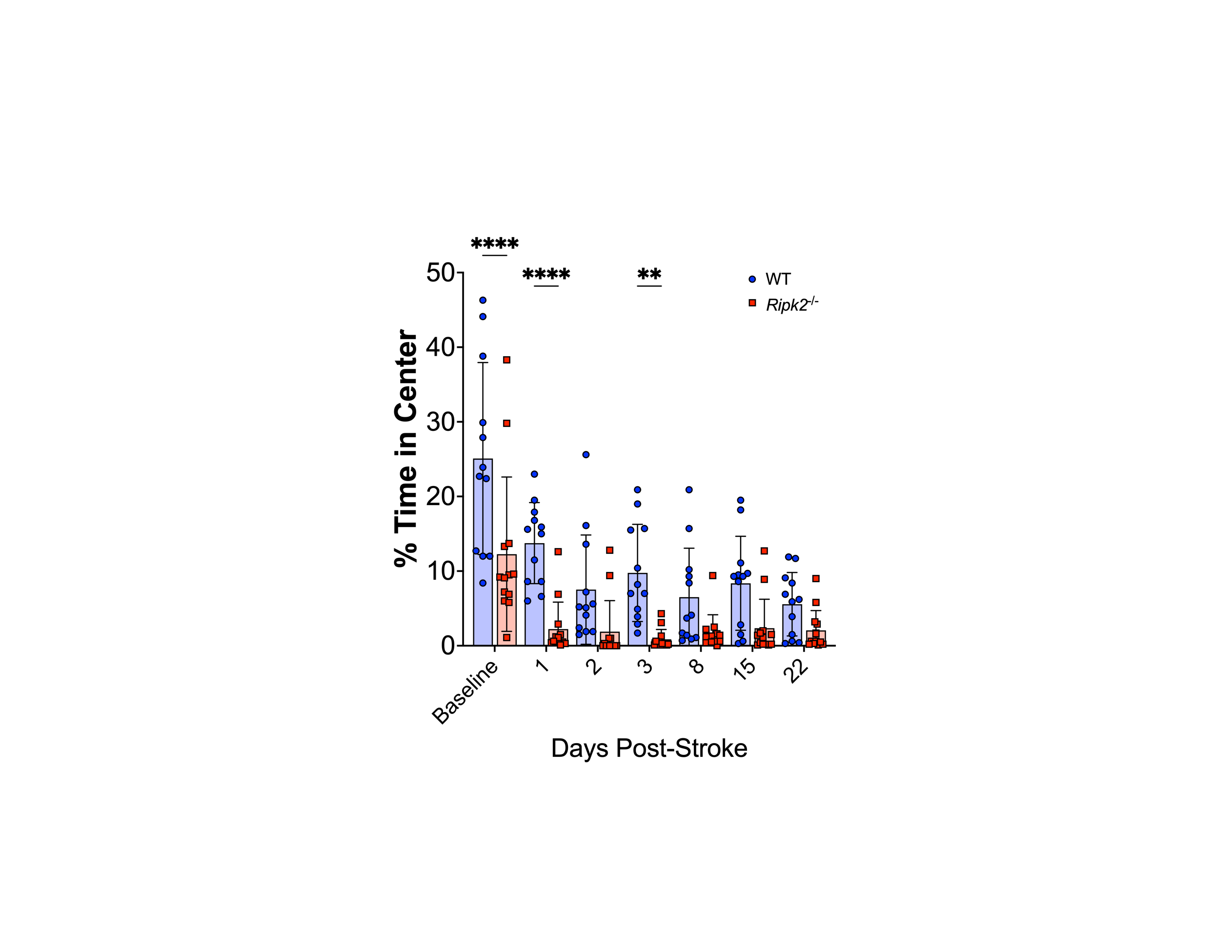


**Supplementary Figure 1: Ripk2-/- mice spend less time in the center of the open field chamber than aged WT control mice.** Quantified percent time in center of the open field arena shows decreased time in center for *Ripk2^-/-^* mice compared to aged WT control mice. Differences detected using two-way repeated measures ANOVA with Šídák’s post-hoc. ** *p* < 0.01, **** *p* < 0.0001.
